# Recent progress in pneumococcal protein vaccines

**DOI:** 10.3389/fimmu.2023.1278346

**Published:** 2023-09-25

**Authors:** Sha Li, Hangeri Liang, Shui-Hao Zhao, Xiao-Yan Yang, Zhong Guo

**Affiliations:** ^1^ Zhuhai Key Laboratory of Basic and Applied Research in Chinese Medicine, School of Bioengineering, Zunyi Medical University, Zhuhai, Guangdong, China; ^2^ Center for Biological Science and Technology, Advanced Institute of Natural Sciences, Beijing Normal University, Zhuhai, Guangdong, China

**Keywords:** *Streptococcus pneumoniae*, Pneumococcal protein vaccine, pneumolysin, pneumococcal surface proteins, pneumococcal histidine triad proteins

## Abstract

Pneumococcal infections continue to pose a significant global health concern, necessitating the development of effective vaccines. Despite the progress shown by pneumococcal polysaccharide and conjugate vaccines, their limited coverage and the emergence of non-vaccine serotypes have highlighted the need for alternative approaches. Protein-based pneumococcal vaccines, targeting conserved surface proteins of *Streptococcus pneumoniae*, have emerged as a promising strategy. In this review, we provide an overview of the advancements made in the development of pneumococcal protein vaccines. We discuss the key protein vaccine candidates, highlight their vaccination results in animal studies, and explore the challenges and future directions in protein-based pneumococcal vaccine.

## 1 Introduction

Pneumococcal infections, caused by the bacterium *Streptococcus pneumoniae* (*S. pneumoniae*), continue to be a major global health burden, particularly affecting vulnerable populations such as young children, the elderly, and immunocompromised individuals (1–4). Pneumococcal diseases encompass a spectrum of illnesses, including pneumonia, meningitis, sepsis, and otitis media, resulting in substantial morbidity and mortality worldwide (2, 4). Antibiotics have traditionally been the primary treatment for *S. pneumoniae* infections; however, the rise of antibiotic-resistant strains has posed significant challenges to effective therapy (5). Therefore, vaccines have emerged as a promising approach to prevent pneumococcal infections (6). Currently, based on a limited number of serotype-specific capsular polysaccharides, there are two types of pneumococcal vaccines are available (7). The first type is the 23-valent pneumococcal polysaccharide vaccines (PPVs) consisted with 23 different capsular polysaccharides. The second type is the multivalent pneumococcal conjugate vaccines (PCVs), which involve polysaccharides conjugated to a carrier protein (8, 9). These vaccines aim to elicit immune responses that protect against pneumococcal infections caused by specific serotypes (10). Immunization with these vaccines targeting the polysaccharide capsule of *S. pneumoniae* has resulted in significant reductions in pneumococcal disease burden, particularly in children (10–12). While PCVs have made remarkable strides in preventing pneumococcal infections, they do have certain limitations (13). Firstly, there are over 90 known pneumococcal serotypes, and PCVs primarily target a limited number of the most common ones. This leaves a significant proportion of pneumococcal strains unaddressed, leading to a phenomenon known as serotype replacement, whereby non-vaccine serotypes emerge and cause infections (14). Furthermore, the polysaccharide antigens used in PCVs have poor immunogenicity in very young children and immunocompromised individuals, limiting the vaccine’s efficacy in these populations (15).

**Figure 1:**
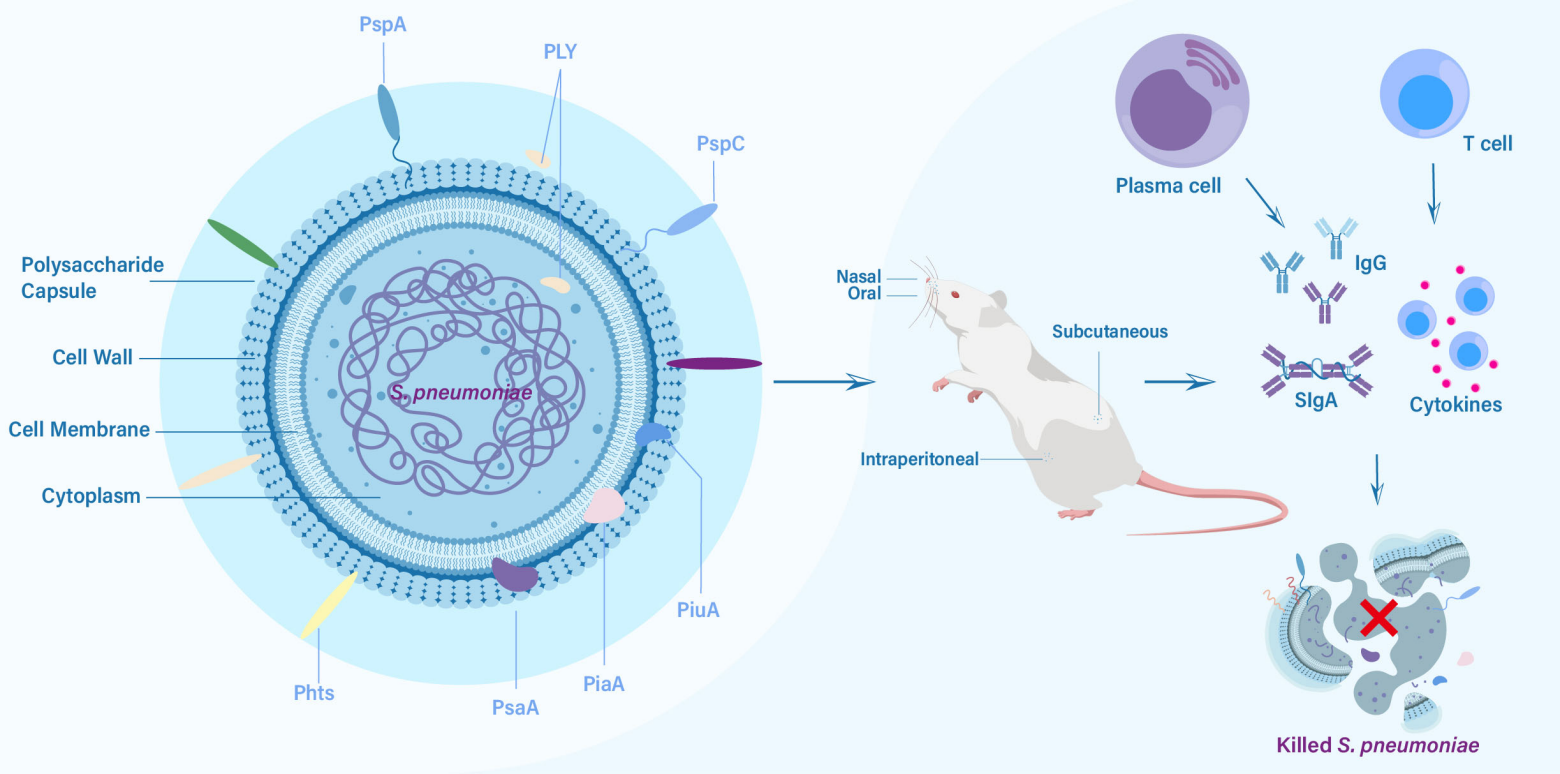
The key protein vaccine candidates of *S. pneumoniae*, routes of vaccination, and elicited immune responses against challenge from *S. pneumoniae*.

Protein-based pneumococcal vaccines have emerged as a promising strategy to address the limitations of polysaccharide-based vaccines (16). Unlike polysaccharide capsules, which exhibit significant antigenic variation among different serotypes, certain surface proteins of *S. pneumoniae* are more conserved and shared across serotypes (17). By targeting these conserved proteins, protein-based vaccines have the capacity to offer broader protection against a wider range of pneumococcal strains, including those not covered by PCVs (18). The development of pneumococcal protein vaccines has been fueled by advancements in understanding the biology and pathogenesis of *S. pneumoniae*. Identification of key virulence factors and surface proteins involved in host-pathogen interactions has facilitated the selection of potential vaccine candidates (19). Pneumolysin (PLY), pneumococcal surface protein A (PspA), pneumococcal surface protein C (PspC), pneumococcal histidine triad proteins (Pht), pneumococcal surface antigen A (PsaA), pneumococcal iron uptake A (PiuA) and pneumococcal iron acquisition A (PiaA) are among the most extensively studied protein candidates, each with unique immunogenic properties and mechanisms of immune protection (**Figure 1**) (16, 20–22). In this review, we aim to comprehensively explore the progress made in pneumococcal protein vaccines, focusing on key protein candidates and future directions. By understanding the advancements and remaining gaps in this field, we can envision a future where protein-based pneumococcal vaccines play a crucial role in reducing the burden of pneumococcal diseases and protecting vulnerable populations.

## 2 Protein candidates for pneumococcal vaccines

### 2.1 Pneumolysin, PLY

PLY is a vital cholesterol-dependent cytolysin produced by *S. pneumoniae* (**Table 1**) (23). With 471 amino acids (53 kDa) and a distinct tertiary structure consisting of four domains, PLY binds to eukaryotic cell membranes’ cholesterol and creates membrane pores, leading to cellular destruction (20). Domains 1, 2, and 3 are essential for oligomerization and pore formation, while domain 4, located in the C-terminal region, facilitates cholesterol binding (20). PLY serves as a critical virulence factor in various stages of pneumococcal disease, including transmission, colonization, and infection (23). It exerts its effects through multiple mechanisms. PLY has the capacity to activate the classical complement pathway, trigger inflammation, induce apoptosis or necroptosis, and directly cause cell toxicity (23, 24). Furthermore, PLY can interact with the mannose receptor C type 1, resulting in the downregulation of inflammation and promoting bacterial survival in the airways (35). Significantly, PLY exhibits a high degree of conservation in its amino acid sequence across different pneumococcal serotypes, making it an attractive target for vaccine development (36). By targeting PLY, vaccines can potentially elicit immune responses that neutralize its cytolytic activity, prevent cellular damage, and limit the spread of pneumococcal infections.

**Table 1:** Localization and function of *Streptococcus pneumoniae* selected virulence proteins.

Protein	Localization	Function	selected references
PLY	Cytoplasm/Cell membrane	Activates the classical complement pathway, triggers inflammation, induces apoptosis or necroptosis, and directly causes cell toxicity	(23, 24)
PspA	Surface protein	Inhibits complement component C3, reduces phagocytosis of pneumococci, binds to lactoferrin	(25, 26)
PspC	Surface protein	Inhibits complement component C3 and factor H, binds to secretory IgA, binds to host cell laminin receptor	(27, 28)
Pht	Surface protein	Inhibits complement component C3, binds zinc ion	(29, 30)
PsaA	Surface protein	Binds to manganese and zinc ion, resists oxidative stress, reduces bacterial adhesion,	(31, 32)
PiuA	Surface protein	Transports ferrichrome iron	(33, 34)
PiaA	Surface protein	Transports heme iron	(33, 34)

It has been well-established that immunizing mice with purified PLY provides protection against highly virulent pneumococci challenges (37). It has reported that murine monoclonal antibodies targeting PLY have demonstrated a reduction in bacterial burden in the lungs and protection against invasive pneumococcal disease (38). Humans naturally develop an antibody response to PLY due to exposure to *S. pneumoniae*. The belief that human antibodies to PLY may be protective is supported by evidence showing that anti-PLY antibodies can delay pneumococcal carriage in high-risk infants and provide protection against pneumococcal infections in healthy individuals (39, 40). Although initial experiments with PLY immunization showed promise as it offered protection against multiple *S. pneumoniae* serotypes, PLY still retained its hemolytic activity in host cells (41). To address this issue, various derivatives of detoxified PLY mutants have been developed, such as recombinant pneumolysoid with the cytolytic functionality removed. This derivative has displayed reactivity to IgGs targeting critical PLY epitopes and has proven successful as an immunogen for specific serotypes in mice and rhesus macaques (38, 42, 43). Further, a self-biomineralized calcium phosphate (CaP)-pneumolysoid nanoparticle vaccine induces bone marrow-derived dendritic cells (BMDCs) and splenocytes production of cytokine, and elicits efficient humoral and cellular immune responses to protect mice from both pneumonia and sepsis infection (44).

Additionally, chemically detoxified PLY derivatives have been explored, inducing an IgG response against PLY without causing tissue damage, as observed in histopathology examinations of host tissues. These derivatives have shown efficacy in protecting against intranasal challenges involving three distinct serotypes (45). Additionally, the protective effects induced by a PLY or pneumolysoid vaccine could potentially be enhanced through the incorporation of other antigens that elicit protection. One such antigen is PspA, which induces high levels of antibodies against each protein and confers protection to mice against invasive challenges, some of which have advanced to phase II trials (21, 46). In addition, immunization mice with pneumolysoid fused with pneumococcal SP0148 has shown to elicit high level of antibody in the serum and effective protection against pneumococcal challenge (47). Moreover, PLY has shown promise as a carrier protein in PCV formulations, especially when combined with CPS. Immunization studies in mice using pneumolysoid conjugated to type 19F CPS have demonstrated a robust and boostable antibody response against both the protein and CPS components. This immunization approach resulted in a high level of protection for infant mice when they were challenged with *S. pneumoniae *(48). Similar positive outcomes have been observed for conjugates of native PLY with type 18C CPS (49). Comparisons between tetravalent pneumolysoid-CPS conjugate vaccines and tetanus toxoid-CPS conjugate vaccines have revealed that pneumolysoid performs at least as well as tetanus toxoid as a carrier protein, and in some cases, such as with type 23F, it has shown superiority (50). These findings highlight the potential of such antigens to elicit a substantial immune response against CPS, as well as an immune response against virulence proteins, thereby offering comprehensive protection against pneumococcal disease in humans. Extensive animal studies and ongoing clinical trials have positioned PLY as a promising candidate for incorporation into multicomponent protein vaccines. The diverse range of detrimental effects exerted by PLY on the host highlights its significant potential as a valuable vaccine target.

### 2.2 Pneumococcal surface protein A, PspA

PspA, one of the extensively studied choline-binding proteins, belongs to the major class of *S. pneumoniae* surface proteins (**Table 1**) (51, 52). It is widely expressed by all capsular serotypes of *S. pneumoniae* and serves as a crucial virulence factor that influences bacterium-host interactions by interfering with the fixation of complement C3 (52–54). PspA consists of three major domains: an α-helical domain at the N-terminus, a proline-rich domain in the central portion, and a choline-binding domain at the C-terminus (55). Its α-helical domain exhibits high variability between serotypes and strains. The sequence diversity of PspA has led to its classification into three families and six clades. Family 1 encompasses clades 1 and 2, family 2 comprises clades 3, 4, and 5, while family 3 includes clade 6 (55). Notably, a significant majority of pneumococcal isolates (ranging from 94 to 99%) belong to PspA families 1 and 2 (56), further highlighting its relevance in the global distribution of pneumococci (57). PspA plays pivotal roles in inhibiting complement component C3 deposition on the pneumococcal surface (25), reducing phagocytosis of pneumococci (58, 59), and providing substantial protection against bactericidal peptides of lactoferrin (26). Given its functional significance, PspA has been explored for over three decades as an immunogen and a potential vaccine candidate (22, 60, 61).

Immunization with recombinant family 1 PspA has demonstrated its immunogenicity in humans, with antibodies generated by PspA offering passive protection to mice against a pneumococcal challenge from serotypes 3, 6A, or 6B (62). Subcutaneous immunization with PspA has proven effective in protecting mice from fatal infections (63), while intranasal immunization with PspA has shown efficacy in protection against nasopharyngeal carriage in an adult mouse carriage model (64, 65). Furthermore, mucosal vaccination with PspA has successfully elicited both mucosal and systemic immune responses (66, 67). Initially, there were concerns that a vaccine targeting PspA might not provide adequate coverage due to its high variability (68). However, recent research has brought attention to the proline-rich domain of PspA, which has been found to be considerably more conserved (60). This discovery suggests that the proline-rich domain could be utilized to target uncommon strains beyond those belonging to family 1 or family 2, potentially enhancing the vaccine’s efficacy and widening its scope of protection (22, 68). Additionally, mice immunization with fusion proteins containing family 1 and family 2 PspA fragments could increase complement deposition and provide protection against pneumococcal infection with strains bearing PspA fragments from both families (69). Furthermore, a fusion protein vaccine combined PspA containing families 1 and 2 with other protein can potentially protect against a wide range of *S. pneumoniae* strains (70). Moreover, a PspA-based trivalent pneumococcal vaccine was formulated and the immunogenicity and protection efficacy were evaluated in macaques, which demonstrated the trivalent vaccine could target all families and clades of PspA and elicited high IgG titers and provided protection against pneumococcal intratracheal challenge (71). PspA has completed a phase I trial (NCT01033409). Further, PspA-based fusion protein vaccine or conjugate vaccine formulations with CPS induce high immune responses and protect mice against invasive challenge (21, 46, 72).

The conjugation of PspA with Vi CPS (*Salmonella typhi* CPS) has shown enhanced anti-PspA responses and elicited a T-cell dependent response to Vi (72). Additionally, immunization with a PspA fusion protein in combination with CpG oligonucleotides and aluminum hydroxide gel provided significant protection against pneumococcal challenge in mice (73). Moreover, intranasal immunization with a PspA-based vaccine, fused with a protein anchor to display PspA on the surface of bacterium-like particles or pneumolysin, efficiently induced both IgG in the serum and IgA in mucosal washes, ultimately providing complete protection against pneumococcal challenge in mice (74, 75). Despite the challenge posed by the variability in PspA, it remains a robust candidate with decades of research backing its potential as a valuable vaccine component.

### 2.3 Pneumococcal surface protein C (PspC)

PspC, also known as CbpA, SpsA, or Hic, is another choline-binding protein that was initially identified due to its homology with PspA (**Table 1**) (76). Similar to PspA, PspC features an N-terminal α-helical domain, a central proline-rich domain, and a choline-binding domain (77). However, a significant difference between the two proteins lies in the complexity of the α-helical domain of PspC, which exists in several distinct alleles with different combinations of functions (78). PspC exhibits binding capabilities to secretory IgA, complement component C3, and complement factor H, contributing to its potential roles in colonization, adherence, and invasion (27, 28). Notably, PspC can inhibit C3 deposition on the bacterial surface and mediate the translocation of pneumococci from the nasopharynx to sterile sites such as the lungs or bloodstream (28, 76). Due to its highly immunogenic nature, anti-PspC antibodies play a significant role in antibody immunity against *S. pneumoniae *(79). Numerous studies have demonstrated that PspC vaccines can induce robust immune responses and provide protection against carriage or invasive challenges in mice (80, 81).

PspC has exhibited promising potential as a vaccine candidate, capable of providing protection as the sole immunogen against pneumococcal infection and carriage (22, 80, 82). Nasal immunization with PspC has been shown to prime the immune system of mice, leading to faster immune responses and a reduction in pneumococcal colonization (83). Subcutaneous immunization with PspC has elicited cross-reactive antibodies with PspA, resulting in mice being protected against pneumococcal sepsis challenges (84). Moreover, intranasal immunization with PspC induced high level of anti-PspC antibodies, and the anti-PspC antiserum from intranasally immunized mice significantly inhibited the adhesion of *S. pneumoniae* to A549 cells (82). Although a study reported that immunization with PspC through nasal or subcutaneous route did not confer protection against specific pneumococcal challenges (85), PspC has shown to produce additive and longer-lasting immune responses and broaden the range of serotypes covered when combined with other pneumococcal immunogens (46, 86). These combination strategies aim to target multiple virulence factors of *S. pneumoniae*, thereby providing a more comprehensive and prolonged immune response against pneumococcal infections (86). Notably, one such combination involved fusing PspC with L460D-pneumolysoid as a fusion protein, which demonstrated broader immunogenicity compared to PspC alone. This fusion protein displayed protection against pneumococcal infections and the possibility of providing additional protection against other meningeal pathogens (87). Additionally, recent studies have identified the NEEK motif of PspC, which has the ability to bind to laminin receptors on the blood-brain barrier, suggesting its potential importance in eliciting protection against fatal pneumococcal infections, particularly in cases of meningitis (22, 88).

Studies investigating PspC-based vaccines in animal models have shown promising results, highlighting their immunogenicity and protective efficacy (22). However, the development of PspC-based vaccines still faces certain challenges that need to be addressed. Ongoing research is focused on optimizing vaccine formulations, identifying the most immunogenic PspC variants, and determining the ideal combination strategies with other antigens. By addressing these challenges, PspC-based vaccines hold great potential for providing effective protection against pneumococcal infections.

### 2.4 Pneumococcal histidine triad proteins, Pht

Pht proteins constitute a group of surface proteins identified in *S. pneumoniae*, comprising four members: PhtA, PhtB, PhtD, and PhtE (89). The discovery of the Pht family was based on the presence of hydrophobic leader sequences, suggesting their localization as cell surface proteins after transport across the cytoplasmic membrane (**Table 1**) (90). These proteins contain a distinctive polyhistidine motif, HXXHXH, which is repeated five times (PhtA, PhtB, and PhtD) or six times (PhtE) within their amino acid sequences (89). Ranging in size from 91.5 to 114.6 kDa, these four proteins exhibit a close relationship at the amino acid sequence level, with identities ranging from 32% to 87%. The N-terminal regions demonstrate the highest similarity among the four proteins, exhibiting 87% identity (89, 90). Pht proteins play a crucial role in the pathogenesis of *S. pneumoniae* infections and are considered multifunctional virulence factors (91). Although their precise functions are not fully understood, they are known to be involved in metal-ion binding, particularly zinc, which is vital for bacterial survival and growth (29). Additionally, Pht has been shown to interact with various host proteins, modulating the host immune response. For instance, Pht has been reported to inhibit the deposition of complement component C3 on the pneumococcal surface through the recruitment of factor H (30). Given their immunogenic properties, Pht has garnered interest as a potential vaccine candidate for non-serotype-dependent prevention of pneumococcal infections.

There is a high degree of protein sequence conservation among various Pht proteins across different serotypes of *S. pneumoniae *(92). Numerous studies have demonstrated the immunogenicity and protective efficacy of all Pht proteins in multiple mouse models of invasive disease, sepsis, and nasopharyngeal colonization when challenged with diverse *S. pneumoniae* strains (93–95). Immunizations with different portions of PhtA, including the N-terminal or C-terminal regions, as well as full-length PhtA, generated high levels of antibody titers (90). Full-length PhtA induced antibodies that protected mice against sepsis challenge with serotype 6A or serotype 6B (90). Immunization with either the N-terminal or C-terminal portion of PhtA also conferred protection against serotype 6B challenge, while only the N-terminal half of PhtA induced a protective response against serotype 6A challenge (90). Similarly, immunization with full-length PhtB also elicited high levels of antibody titers and protected mice against challenge with serotype 6B (90). Additionally, immunization with recombinant PhtB provided protection against serotype 3 intranasal pneumococcal challenge (96). Furthermore, immunization of mice with PhtD resulted in the highest levels of antibody titers among all Pht-based vaccines and protected mice against challenge with serotype 3 and 4. Intranasal immunization with PhtD induced robust serum antibody and CD4 Th1-biased immune memory, providing protection against pneumococcal colonization (97). Recent studies using PhtD and C-terminal fragment of PhtD with alum or outer-membrane vesicles as adjuvants elicited significantly high levels of antibodies and conferred protection against pneumococcal challenge (93, 98, 99). In addition, anti-PhtD antibodies were shown to protect against *S. pneumoniae* through complement- and macrophage-dependent opsonophagocytosis (100). Among the Pht proteins, PhtD is considered the most suitable candidate due to its superior efficacy in a nasopharyngeal colonization model and its high level of conservation among pneumococcal strains (92). PhtD has undergone phase I trials (NCT01767402 and NCT01444001) and failed in an otitis media clinical trial (101, 102). Additionally, Pht-based vaccines have been tested in combination with other protein antigens, such as PspA and PLY, to explore synergistic effects and broaden the spectrum of serotype coverage (43, 103). PhtD conjugated with PLY elicited significant protection that have made it up to phase II trials (Clinical Trial Number, NCT01262872 and NCT00896064). Despite these promising results, challenges remain in the development of Pht-based vaccines. Further studies are needed to identify the most immunogenic epitopes within Pht proteins and to optimize vaccine formulations that induce protective immune responses against multiple pneumococcal strains.

### 2.5 Pneumococcal surface antigen A, PsaA

PsaA is an important protein found on the surface of *S. pneumoniae*, which plays a crucial role in the pathogenesis of pneumococcal infections (104). This surface-exposed lipoprotein has an approximate molecular weight of 37 kDa and is involved in vital functions, including oxidative stress resistance, bacterial adhesion, and acquisition of essential metal ions, particularly manganese and zinc, imperative for pneumococcal growth and viability (**Table 1**) (31, 32, 105, 106). By scavenging these metal ions from the host environment, PsaA aids the bacterium in evading the host immune response and establishing infections (107). Notably, PsaA exhibits a high degree of conservation across various serotypes of *S. pneumoniae*, making it an attractive target for vaccination strategies aimed at providing broad protection against multiple strains (108).

Utilized as an immunogen, PsaA has been shown to induce a strong immune response, making it an important target for eliciting protective immune responses through vaccination (37). Parenteral immunization of mice with PsaA using adjuvants has yielded substantial protection against challenge from type 3 pneumococcal strain WU2 (109). Similarly, oral or intranasal administration of PsaA has led to elevated titers of IgG anti-PsaA antibodies in serum and IgA antibodies in mucosal sites. These responses have correlated with a notable reduction in nasopharyngeal colonization following intranasal exposure to *S. pneumoniae *(110). However, in other study immunization with PsaA elicited antibody response, which cannot effectively protect mice from challenge with *S. pneumoniae *(111). This prompted the exploration of combinatory approaches, wherein PsaA was fused with other protein antigens to amplify immune responses and synergistic effects. Immunization with PsaA fused with PspA, for instance, triggered heightened antibody levels against both PsaA and PspA. This conferred reduced *S. pneumoniae* levels in the bloodstream and lungs, ultimately shielding against fatal challenges with the pathogen (112). Moreover, the fusion of PsaA with B lymphocyte stimulator has exhibited potent immune stimulation, marked by heightened serum antibodies specific to PsaA and elevated cytokine levels (113). Additionally, immunization with PasA effectively provides protection when combined with adjuvants in animal challenge (114). Notably, PsaA has also found utility as a carrier protein in glycoconjugate vaccines, displaying protective efficacy in animal models (115). These finding underscore that PsaA represents an appealing candidate for incorporation into pneumococcal vaccines, offering the potential to enhance protection against an extensive array of pneumococcal infections and reduce the burden of disease worldwide. PsaA included vaccine formulations with other proteins have completed or be active in clinical trials (NCT00873431, NCT03803202, and NCT04525599).

### 2.6 Iron transport lipoproteins, PiuA and PiaA

Pneumococcal iron uptake A (PiuA) and pneumococcal iron acquisition A (PiaA) are two important iron transport lipoproteins found in *S. pneumoniae*, which are part of the ATP-binding cassette (ABC) transporter system, a common mechanism used by bacteria to transport essential nutrients across the cell membrane (**Table 1**) (33, 116). These proteins play a critical role in the uptake of iron from the host environment and its subsequent utilization within the bacterial cell (117, 118). PiuA and PiaA play roles in iron acquisition, with PiuA responsible for transporting ferrichrome iron and PiaA involved in heme iron transport (33, 34). Given their essential role in iron uptake, PiuA and PiaA have been explored as potential targets for the development of antibacterial strategies. Disruption of these proteins or interference with their iron-binding capabilities could potentially impair bacterial survival and growth (118).

Additionally, PiuA and PiaA have undergone investigation as potential vaccine candidates due to their surface exposure, high conservation and immunogenic properties (116, 119, 120). Immunizing mice with recombinant PiuA and PiaA has led to the generation of robust antibody titers, resulting in enhanced complement-independent and -dependent opsonophagocytosis (121). This immune response has been shown to confer protection against systemic challenges posed by *S. pneumoniae *(119). Moreover, mucosal immunization of mice with PiuA and PiaA has demonstrated the induction of specific antibody responses in both serum and respiratory secretions. This antibody-mediated immune reaction proved effective in safeguarding mice against intranasal challenges from *S. pneumoniae *(120). In addition, serum antibody to PiuA and PiaA from patients with pneumococcal septicaemia was significantly higher in convalescent-phase than acute-phase, and cross-reacted in different serotypes (116). Further evidence of the immunogenicity of PiuA and PiaA comes from the detection of anti-PiuA and anti-PiaA antibodies in healthy seven-month-old infants, indicating that these proteins can elicit an immune response from a very early age (116). However, there are no clinical trials involving PiuA or PiaA.

### 2.7 Whole-cell vaccines

Whole-cell vaccines are type of vaccines that use the entire cell of the bacterium *S. pneumoniae* and present all protein antigens without the need for individual protein purification to stimulate the immune system. Whole-cell vaccines can be developed from killed or live-attenuated whole-cell or by using genetically modified strains of pneumococcus to ensure safety while retaining immunogenicity. These vaccines approach aim to induce both humoral and cellular protective immune response against multiple antigens of the bacterial cell in animal models.

Killed whole-cell vaccines (WCVs) are typically composed of inactivated or killed *S. pneumoniae* cells. These vaccines can include a variety of strains or serotypes of the bacteria, allowing them to cover a broader spectrum of pneumococcal infections. A WCV was developed by deleting the autolysin gene (*lytA*) in *S. pneumoniae* strain RX1 and killed using ethanol. The vaccine was administered via intranasal route to elicit effective prevention of nasopharyngeal colonization with serotype 6B in mice and confer protection against illness and death in rats with serotype 3 strain (122). In addition, a RM200 WCV was constructed by replacing entire *lytA* gene with a kanamycin resistance gene in strain RX1E with a detoxified PLY mutation (123). The RM200 WCV was inactivated using beta-propiolactone and exhibited strong protective effects against nasopharyngeal colonization by serotype 6B strain and activated IL-17A priming (123, 124).

Live attenuated WCVs contain weakened or attenuated strains of *S. pneumoniae*. These strains are modified so that they can still replicate within the body but are less virulent, causing only mild or no disease symptoms. A live attenuated WCV was achieved through the attenuation of *S. pneumoniae* D39, wherein the *pep27* gene was removed (125). Mice immunized intranasally with the WCV exhibited high level of IgG and serotype-independent protection against lethal intranasal challenge (125). Additionally, to enhance the safety of this WCV, *comD* gene was also removed to constructed a WCV of *Δpep27ΔcomD*. Immunization of mice with *Δpep27ΔcomD* significantly increased the survival time after heterologous challenge and diminished colonization levels of independent of serotype, which indicated that the WCV of *Δpep27ΔcomD* appears to be a highly feasible and safe vaccine against pneumococcal infections (126). These vaccines are currently in clinical trial phase 1/2 (NCT02097472).

## 3 Discussion

The progress in pneumococcal protein vaccines represents a significant advancement in the field of vaccine development against *S. pneumoniae* infections. Protein-based vaccines offer several advantages over traditional capsular polysaccharide-based vaccines, including the potential for broader serotype coverage and the ability to target conserved surface proteins that play critical roles in pneumococcal pathogenesis. Numerous protein candidates have been investigated for their potential as vaccine antigens, including PLY, PspA, PspC, Pht, PsaA, PiuA and PiaA. These proteins have shown promising immunogenicity and protective efficacy in animal studies, with some candidates demonstrating the ability to induce immune responses that inhibit bacterial adherence, colonization, and invasion. Furthermore, several proteins have been evaluated in early clinical trials, providing insights into their safety and immunogenicity in humans. The diversity of protein antigens under investigation highlights the multifaceted nature of pneumococcal pathogenesis and the need for a comprehensive vaccine approach. Combining multiple protein antigens in vaccine formulations, either as multicomponent vaccines or through the use of carrier proteins, holds the potential to broaden serotype coverage and enhance the protective immune response. However, challenges remain in the development of pneumococcal protein vaccines. The high degree of antigenic variability among pneumococcal strains necessitates the identification of conserved epitopes and the design of vaccines that provide broad protection against diverse serotypes. Optimization of vaccine formulations, including the selection of appropriate adjuvants and delivery systems, is crucial to ensure optimal immune responses and long-term efficacy. Furthermore, the evaluation of protein-based vaccines in larger-scale clinical trials is necessary to assess their efficacy in preventing pneumococcal infections and reducing disease burden. Continued surveillance and monitoring of pneumococcal strains are essential to detect any potential serotype replacement or emergence of new strains that may impact vaccine effectiveness.

## Author contributions

SL: Supervision, Writing – original draft. HL: Writing – original draft. S-HZ: Writing – original draft. X-YY: Supervision, Writing – review & editing. ZG: Supervision, Writing – review & editing.
